# Dynamic response of the intestinal microbiome to *Eimeria maxima-*induced coccidiosis in chickens

**DOI:** 10.1128/spectrum.00823-24

**Published:** 2024-09-09

**Authors:** Jing Liu, Jiaqing Guo, Melanie A. Whitmore, Isabel Tobin, Dohyung M. Kim, Zijun Zhao, Guolong Zhang

**Affiliations:** 1 Department of Animal and Food Sciences, Oklahoma State University, Stillwater, Oklahoma, USA; Chengdu University, Chengdu, Sichuan, China

**Keywords:** coccidiosis, *Eimeria*, microbiota, intestinal inflammation, chickens

## Abstract

*Eimeria maxima* is a major cause of coccidiosis in chickens and a key predisposing factor for other economically significant diseases such as necrotic enteritis. However, a detailed understanding of the intestinal microbiome response to *E. maxima* infection is still lacking. This study aimed to comprehensively investigate the dynamic changes of the intestinal microbiome for 14 days post-infection (dpi) with *E. maxima*. Bacterial 16S rRNA gene sequencing was performed with the ileal and cecal digesta collected from mock and *E. maxima-*infected chickens at the prepatent (3 dpi), acute (5 and 7 dpi), and recovery phases (10 and 14 dpi) of infection. Although no notable changes were observed at 3 dpi, significant alterations of the microbiota occurred in both the ileum and cecum at 5 and 7 dpi. By 14 dpi, the intestinal microbiota tended to return to a healthy state. Notably, *Lactobacillus* was enriched in response to *E. maxima* infection in both the ileum and cecum, although individual *Lactobacillus*, *Ligilactobacillus*, and *Limosilactobacillus* species varied in the temporal pattern of response. Concurrently, major short-chain fatty acid-producing bacteria, such as *Faecalibacterium*, were progressively suppressed by *E. maxima* in the cecum. On the other hand, opportunistic pathogens such as *Escherichia*, *Enterococcus*, and *Staphylococcus* were significantly enriched in the ileum during acute infection.

**IMPORTANCE:**

We have observed for the first time the dynamic response of the intestinal microbiota to *Eimeria maxima* infection, synchronized with its life cycle. Minimal changes occur in both the ileal and cecal microbiota during early infection, while significant alterations coincide with acute infection and disruption of the intestinal mucosal lining. As animals recover from coccidiosis, the intestinal microbiota largely returns to normal. *E. maxima*-induced intestinal inflammation likely creates an environment conducive to the growth of aerotolerant anaerobes such as *Lactobacillus*, as well as facultative anaerobes such as *Escherichia*, *Enterococcus*, and *Staphylococcus*, while suppressing the growth of obligate anaerobes such as short-chain fatty acid-producing bacteria. These findings expand our understanding of the temporal dynamics of the microbiota structure during *Eimeria* infection and offer insights into the pathogenesis of coccidiosis, supporting the rationale for microbiome-based strategies in the control and prevention of this condition.

## INTRODUCTION

Coccidiosis, caused by various species of *Eimeria*, remains one of the most economically impactful diseases in poultry, causing annual losses exceeding $13 billion in global broiler production (1). Among the causative agents, *Eimeria maxima* stands out as a major cause of coccidiosis, causing damages to small intestinal lining and disruption of microbiota balance, which subsequently leads to diarrhea, reduced growth performance, and increased mortality in affected chickens (2, 3). Upon infection, the parasites undergo multiple rounds of asexual reproduction in the epithelial cells of the jejunum and ileum within the first 3–4 days (2, 4). Subsequently, sexual differentiation and fertilization occur between 5 and 7 days, resulting in the production of oocysts, which are subsequently shed in feces and become infective upon sporulation (2, 4). Completion of the entire life cycle of *E. maxima* takes 6–7 days, coinciding with the destruction of intestinal mucosal membrane, the appearance of intestinal lesions, and acute fecal shedding of the oocysts (5). Surviving chickens gradually recover from coccidiosis beyond 10 days post-infection (dpi).

The microbiota in the chicken gastrointestinal tract (GIT) comprises a diverse population of bacteria, fungi, archaea, protozoa, and viruses, with bacteria being the predominant group (6, 7). Different species of lactic acid bacteria such as *Lactobacillus, Ligilactobacillus*, and *Limosilactobacillus* dominate the upper GIT, while short-chain fatty acid (SCFA)-producing bacteria, such as *Faecalibacterium,* are abundant in the lower GIT of chickens (6, 7). A balanced intestinal microbiota confers benefits to the host by facilitating the digestion and fermentation of indigestible feed compounds, reducing the colonization of pathogenic microbes, regulating the development and maturation of the host immune system, and maintaining the barrier integrity of intestinal epithelium (8). However, a disturbance in the microbiota balance known as dysbiosis has been implicated to the pathogenesis of both intestinal and extra-intestinal disorders (6–8).

Despite numerous studies investigating the microbiota changes in response to *Eimeria* infections, most have focused on the cecal microbiota shifts in response to *Eimeria tenella* or a mixture of *Eimeria* species (9, 10). No research has explored temporal changes in the intestinal microbiota during coccidiosis. Therefore, this study aimed to characterize alterations in both ileal and cecal microbiota following *E. maxima* infection over a 14-day period, extending beyond a single life cycle and into the recovery phase of the parasites. An in-depth understanding of the temporal dynamics of the intestinal microbiome in response to *Eimeria* infection is crucial for elucidating coccidiosis pathogenesis.

## RESULTS

### Body weight changes in response to *E. maxima* infection

**Fig 1:**
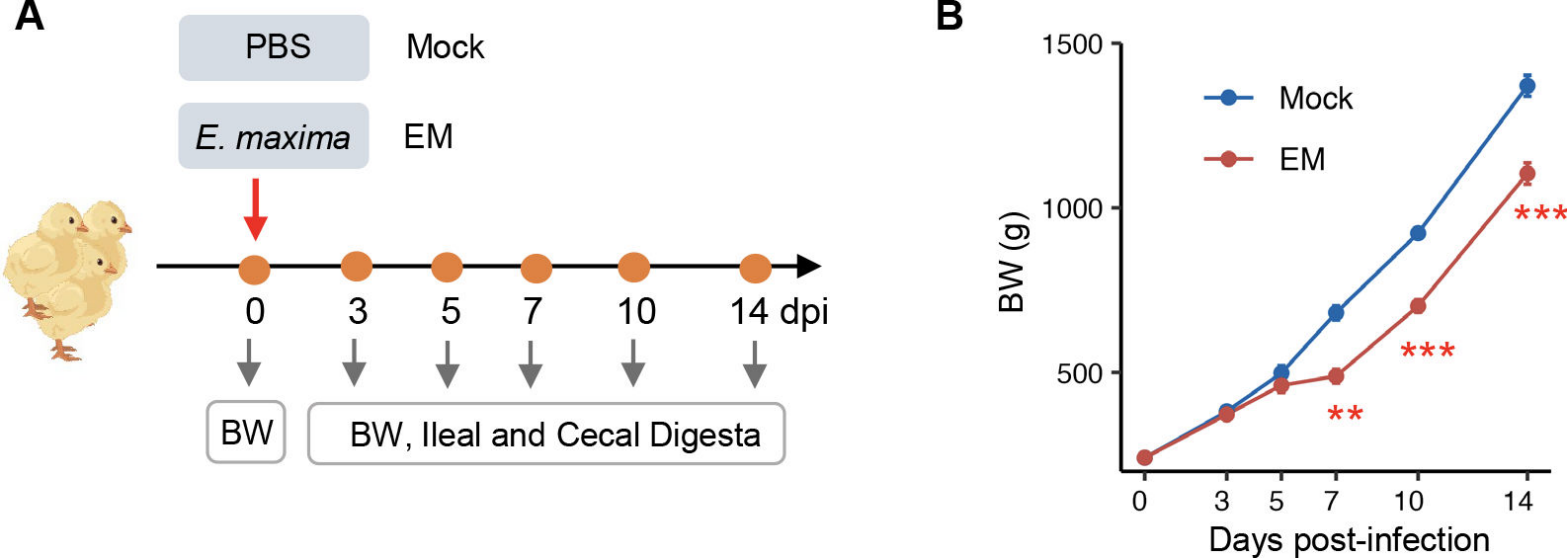
Experimental design and growth performance of chickens following *E. maxima* (EM) infection. (**A**) A total of 120 male Cobb broilers were randomly allocated to two groups on the day of the hatch. On day 10, chickens were either orally challenged with 2 × 10^4^ sporulated oocysts of *E. maxima* M6 or mock-infected with an equal volume of saline. At 3, 5, 7, 10, and 14 dpi, 10 chickens from each group were randomly selected, weighed, and euthanized to collect ileal and cecal digesta samples. (**B**) Body weight (BW) of chickens in the mock and EM-infected groups was monitored over a 14-day period. Data are presented as means ± SEM. **P < 0.01 and ***P < 0.001, relative to the mock group, as determined by Student’s *t*-test.

To evaluate the temporal response of intestinal microbiota to *E. maxima* infection, 10-day-old Cobb broiler chickens were either mock-infected with saline or infected with *E. maxima* to induce coccidiosis. The development of coccidiosis was confirmed by clinical signs such as lethargy, bloody diarrhea, and ruffled feathers exclusive to the infected group. Ten animals from each group were randomly selected, weighed at 3, 5, 7, 10, and 14 dpi, and euthanized for the collection of ileal and cecal digesta for further microbiome analysis (Fig. 1A). While no discernible changes in body weight were observed between mock and *E. maxima*-infected chickens from 0 to 5 dpi, a significant reduction in body weight was evident in infected chickens beginning at 7 dpi (488.8 g vs 681.5 g, *P* = 0.002), which persisted through 14 dpi (1,104.6 g vs 1,371.3 g, *P* < 0.0001) (Fig. 1B). Notably, no mortality was observed following *E. maxima* infection, presumably due to the relatively low dose administered.

### Ileal microbiome dynamics in response to *E. maxima* infection

To elucidate the temporal dynamics of the intestinal microbiome following *E. maxima* infection, bacterial DNA was isolated from the ileal and cecal digesta of mock and *E. maxima*-infected chickens and subjected to 16S rRNA gene sequencing. Following quality control, 12,690,198 high-quality sequencing reads were obtained with an average of 63,451 ± 5,751 sequences per sample for 100 samples. After denoising and removal of amplicon sequence variants (ASVs) present in less than 5% of samples, 453 and 501 ASVs were identified in the ileum and cecum, respectively.

**Fig 2:**
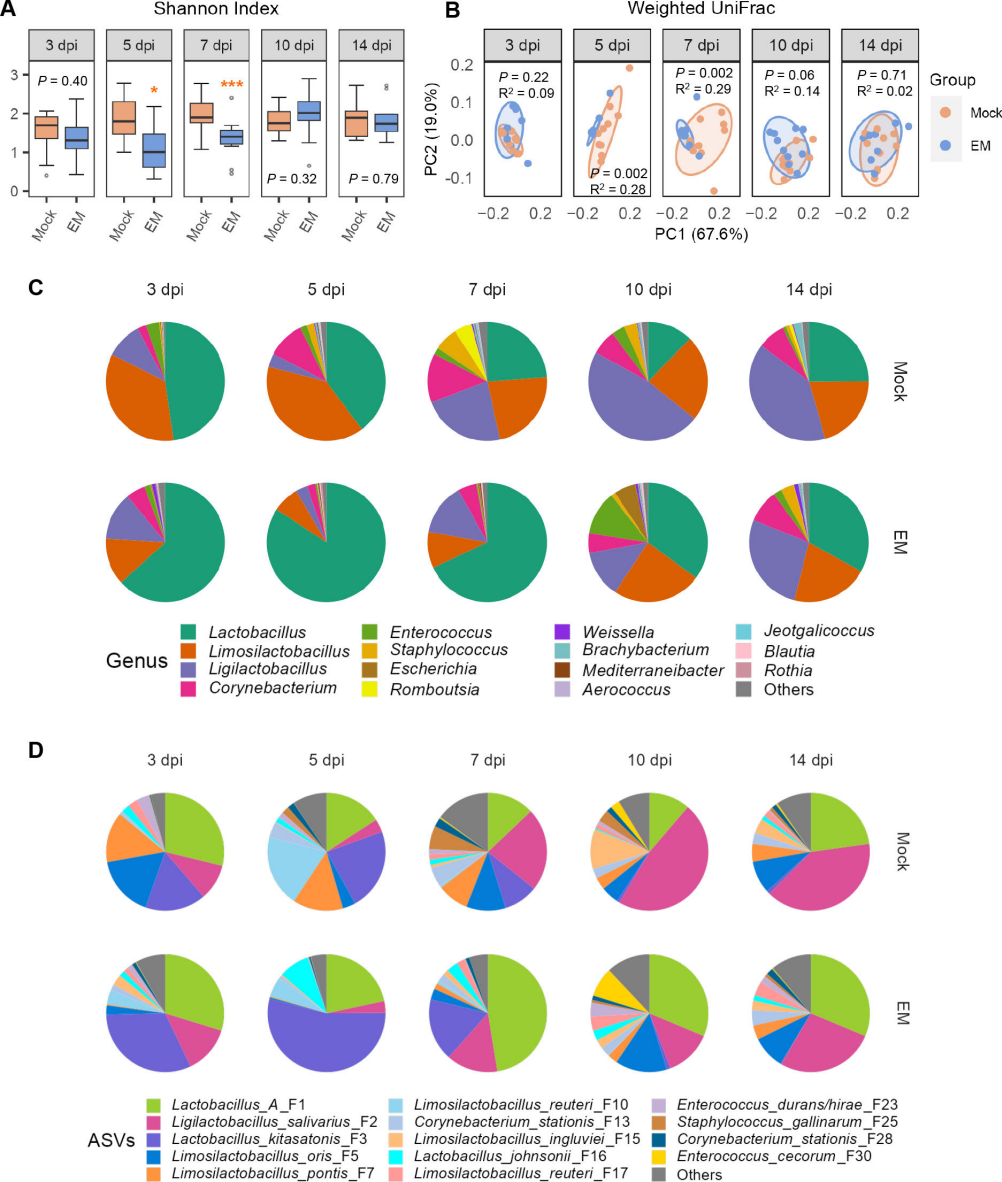
Diversity and composition of the ileal microbiota in response to *E. maxima* (EM) infection. The ileal digesta samples were randomly collected from 10 chickens in either the mock or EM group at each of the five different days post-infection, followed by 16S rRNA gene sequencing and data analysis. (**A**) Shannon index shown in box and whisker plots. Each box indicates the median, 25th, and 75th percentiles, while whiskers extend to the 1.5 interquartile range. *P < 0.05 and ***P < 0.001 as determined by Mann-Whitney *U* test. (**B**) Principal coordinates analysis plots displaying weighted UniFrac distances, with each dot representing an individual ileal digesta sample. Statistical significance was determined using PERMANOVA with 999 permutations. (**C**) Average relative abundances (%) of the top 15 bacterial genera in the ileum across five different dpi. (**D**) Average relative abundances (%) of the top 15 bacterial ASVs in the ileum across five different dpi.

In the ileum, *E. maxima* infection failed to impact the Shannon index until 5 dpi, and a significant decline in the Shannon index (*P* < 0.05) (Fig. 2A) and evenness (Fig. S1A) was noted at 5 and 7 dpi. However, this reduction was temporary, with both the Shannon index and evenness recovering to healthy levels by 10 dpi, although the richness of the ileal microbiota, as indicated by observed ASVs, was not altered throughout the infection (Fig. S1B). Additionally, *E. maxima* infection induced a transient shift, as evidenced by weighted UniFrac (Fig. 2B) and unweighted UniFrac distances (Fig. S1C), in the ileal microbiota at 5 and 7 dpi, with no significant differences observed at other time points. These results indicate that the ileal microbiota composition remained largely unchanged at 3 dpi but underwent a pronounced yet transient shift between 5 and 7 dpi, before returning to normal.

Compositionally, *Lactobacillus*, *Limosilactobacillus*, and *Ligilactobacillus* were the three predominant genera in both the mock and *E. maxima*-infected groups across all time points, collectively accounting for 69.3%–94.7% of the total bacterial population in the ileum (Fig. 2C; Table S1). Among the major *Lactobacillus* species that include *Lactobacillus kitasatonis* (F3) and *Lactobacillus johnsonii* (F16), the most prevalent ASV in the ileum belonged to Group A *Lactobacillus* (F1), which consists of highly related species such as *Lactobacillus acidophilus, Lactobacillus crispatus, Lactobacillus gallinarum, and Lactobacillus amylovorus* (11) that cannot be distinguished by sequencing the V3–V4 region of the bacterial 16S rRNA gene. *Ligilactobacillus* was primarily represented by *Ligilactobacillus salivarius* (F2), while *Limosilactobacillus* predominantly comprised four species, namely *Limosilactobacillus oris* (F5), *Limosilactobacillus pontis* (F7), *Limosilactobacillus reuteri* (F10 and F17), and *Limosilactobacillus ingluviei* (F15) (Fig. 2D). Notably, *E. maxima* infection caused obvious fluctuations among these major bacterial taxa (Fig. 2C and D).

**Fig 3:**
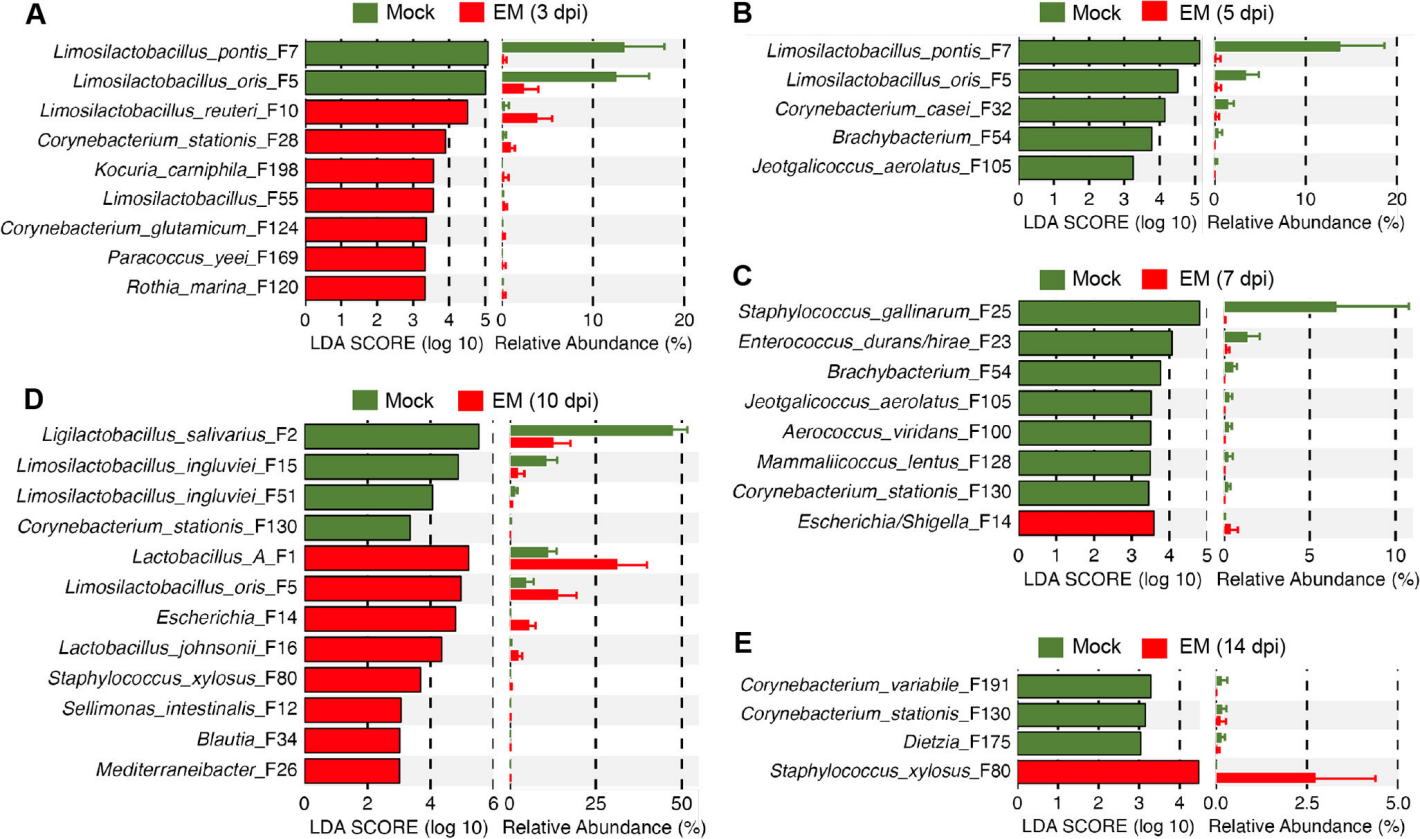
Differential enrichment of ileal bacteria in response to *E. maxima* (EM) infection. The ileal digesta samples were randomly collected from 10 chickens in either the mock or EM group at each of the five different days post-infection, followed by 16S rRNA gene sequencing and data analysis. LEfSe analysis was performed with the top 50 ileal bacterial ASVs between the mock and EM groups at 3 dpi (**A**), 5 dpi (**B**), 7 dpi (**C**), 10 dpi (**D**), and 14 dpi (**E**). The cut-off threshold was set at *P* < 0.05 and LDA score ≥ 3.0. Each panel shows the LDA score of differentially enriched ASVs on the left, while the relative abundances of these ASVs in the mock and EM groups are displayed on the right.

Linear discriminant analysis (LDA) effect size (LEfSe) (12) was subsequently conducted with the top 15 genera and the top 50 ASVs, resulting in the identification of a number of differentially enriched bacteria in response to *E. maxima* infection (Fig. S2; Fig. 3). Among the most prevalent lactic acid bacteria in the ileum*, Limosilactobacillus* exhibited a significant decline in *E. maxima*-infected chickens at 3 and 5 dpi, while *Lactobacillus* showed enrichment at 5, 7, and 10 dpi and *Ligilactobacillus* decreased significantly at 10 dpi (Fig. S2). No differential enrichment of major lactic acid bacteria was observed at 14 dpi, following the recovery of the animals from coccidiosis.

At the species level, both *L. pontis* (F7) and *L. oris* (F5) showed significant reductions at both 3 and 5 dpi, while two other *Limosilactobacillus* species (F10 and F55) were enriched in the *E. maxima* group at 3 dpi (Fig. 3A and B). Additionally, *L. salivarius* (F2) and two strains of *L. ingluviei* (F15 and F51) were reduced at 10 dpi following *E. maxima* infection, while three other lactic acid bacteria, namely group A *Lactobacillus* (F1), *L. oris* (F5), and *L. johnsonii* (F16), were enriched (Fig. 3D). Among those commensal bacteria with pathogenic potential, *Escherichia* (F14) increased at 7 and 10 dpi, while *Enterococcus* (F23) decreased at 7 dpi (Fig. 3C and D). Moreover, *Staphylococcus gallinarum* (F25) reduced in *E. maxima*-infected chickens at 7 dpi (Fig. 3C), while *Staphylococcus xylosus* (F80) became enriched in *E. maxima*-infected chickens at 10 dpi (Fig. 3E).

**Fig 4:**
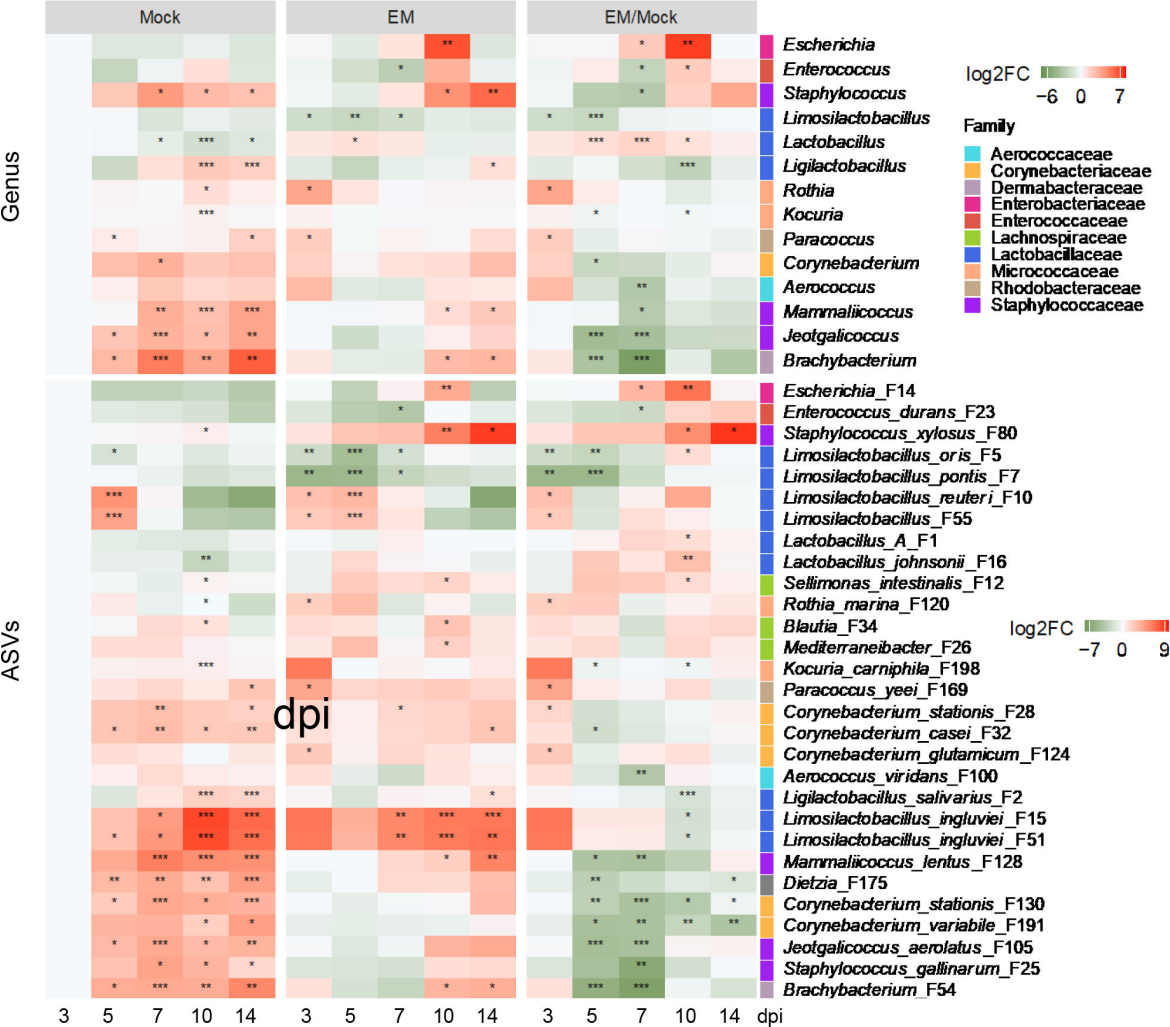
Dynamic patterns of differentially enriched ileal bacterial genera (top) and ASVs (bottom) in response to *E. maxima* (EM) infection. The left two panels of the heatmap depict log_2_ transformations of the fold changes of the differentially enriched bacteria in the ileum of mock and EM-infected chickens across five different days post-infection, relative to mock-infected animals at 3 dpi. *P < 0.05, **P < 0.01, and ***P < 0.001, relative to mock-infected animals at 3 dpi as determined by the Kruskal-Wallis test and *post hoc* Dunn’s test with Benjamini-Hochberg adjustment. The right panel illustrates log_2_ transformations of EM/mock fold differences of individual bacteria on each sampling day for the left two panels. *P < 0.05, **P < 0.01, and ***P < 0.001 between the mock and EM-infected animals on each sampling day as determined by Mann-Whitney *U* test with Benjamini-Hochberg adjustment.

Distinct temporal patterns of differential enrichment were evident among ileal bacteria in *E. maxima*-infected chickens (Fig. 4; Table S1). Among dominant lactic acid bacteria, *Lactobacillus* exhibited a progressive enrichment, peaking at 7 dpi before gradually returning to baseline levels by 14 dpi. Conversely, *Limosilactobacillus* and *Ligilactobacillus* were suppressed by *E. maxima*, albeit with differing kinetics. *Limosilactobacillus* decreased during early infection between 3 and 5 dpi, while *Ligilactobacillus* exhibited a pronounced decline between 7 and 10 dpi (Fig. 4). Individual lactic acid bacterial species showed even more complex patterns of differential enrichment in response to *E. maxima* infection. Group A *Lactobacillus* (F1) and *L. johnsonii* (F16) increased between 5 and 10 dpi and largely returned to normal levels by 14 dpi; however, *E. maxima* had no significant impact on *Lactobacillus kitasatonis* (F2) in the ileum throughout 2 weeks of infection (Table S1). *L. salivarius* (F2) was the only *Ligilactobacillus* species detected in the ileum and showed a transient significant decline at 10 dpi. Among several *Limosilactobacillus* species in the ileum, *L. oris* (F5) and *L. pontis* (F7) showed an obvious decrease during early infection (3–5 dpi) but gradually restored to healthy levels, whereas *L. reuteri* (F10 and F55) and two *L. ingluviei* strains (F15 and F51) were enriched early at 3 dpi but gradually returned to normal levels.

*Escherichia* (F14) proliferated at 7 dpi, peaked at 10 dpi, and subsided completely by 14 dpi, while *Enterococcus* (F23) was only enriched during the recovery phase of infection. Two *Staphylococcus* species also showed markedly different enrichment patterns. *S. gallinarum* (F25) diminished gradually in response to *E. maxima* infection, reaching its lowest abundance at 7 dpi, while *S. xylosus* (F80) progressively increased over the entire course of infection, peaking at 10 and 14 dpi. Several other *Staphylococcaceae* members, such as *Mammaliicoccus lentus* (F128) and *Jeotgalicoccus aerolatus* (F105), as well as several *Corynebacterium* species (F28, F32, F124, F130, and F191), experienced a peak suppression between 5 and 7 dpi during acute *E. maxima* infection (Fig. 4).

### Cecal microbiome dynamics in response to *E. maxima* infection

**Fig 5:**
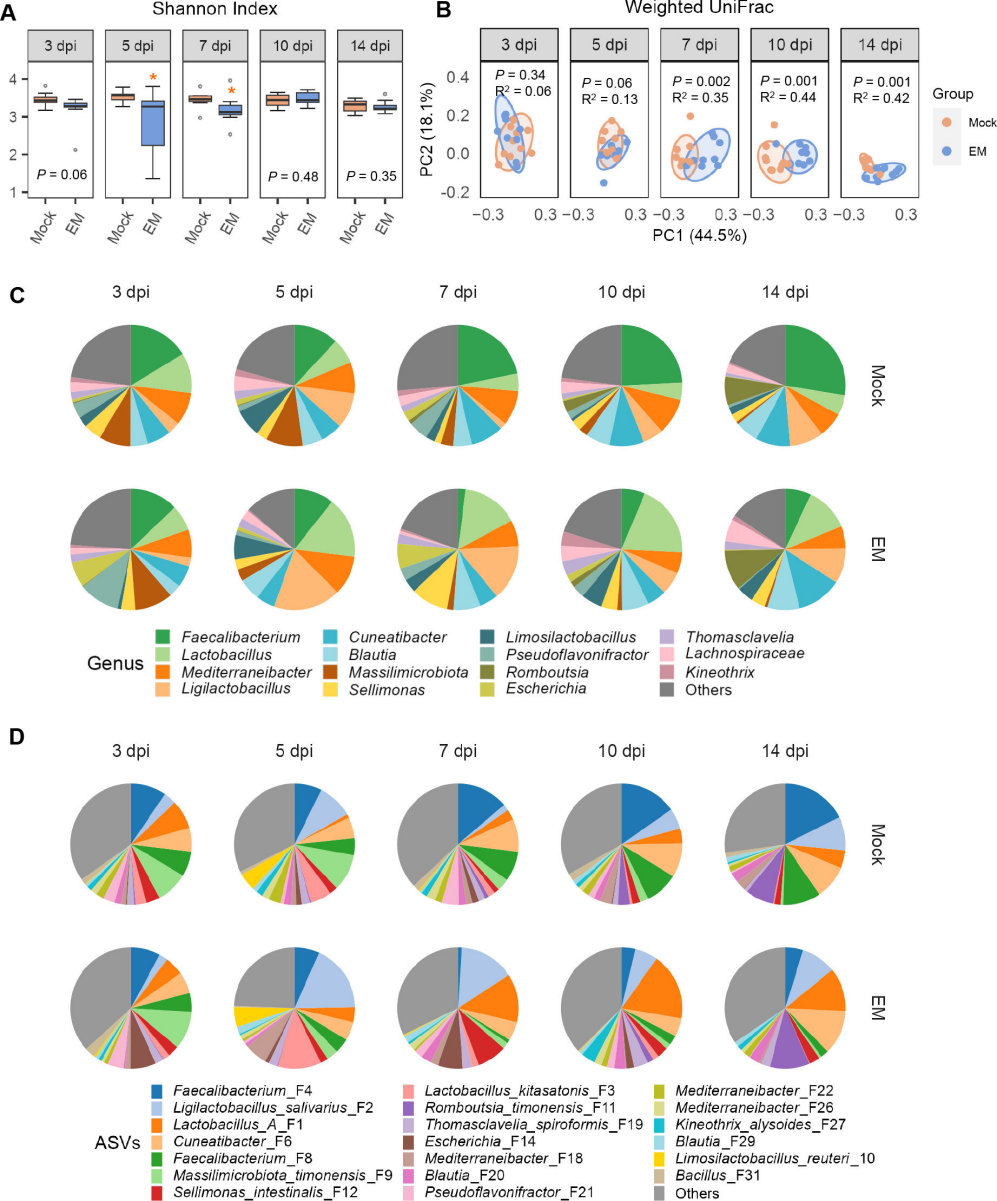
Diversity and composition of the cecal microbiota in response to *E. maxima* (EM) infection. The cecal digesta were randomly collected from 10 chickens in either the mock or EM group at each of the five different days post-infection, followed by 16S rRNA gene sequencing and data analysis. (**A**) Shannon index shown in box and whisker plots. Each box indicates the median, 25th, and 75th percentiles, while whiskers extend to 1.5 interquartile range. *P < 0.05 and ***P < 0.001 as determined by Mann-Whitney *U* test. (**B**) Principal coordinates analysis plots displaying weighted UniFrac distances, with each dot representing an individual cecal digesta sample. Statistical significance was determined using PERMANOVA with 999 permutations. (**C**) Average relative abundances (%) of the top 15 bacterial genera in the ileum across five different dpi. (**D**) Average relative abundances (%) of the top 15 bacterial ASVs in the ileum across five different dpi.

Similar to the changes observed in the ileum, infection with *E. maxima* led to a significant decrease in the Shannon index in the cecum at 5 and/or 7 dpi, although this impact diminished over time (Fig. 5A). Additionally, the number of observed ASVs significantly reduced at 5 and 7 dpi (Fig. S3A), and evenness experienced a significant decline at 5 dpi (Fig. S3B). Furthermore, *E. maxima* induced a significant shift in β-diversity in the cecum at 7 dpi, as evidenced by weighted UniFrac (Fig. 5B) and unweighted UniFrac distances (Fig. S3C). However, unlike the ileal microbiota that tended to normalize beyond 7 dpi following *E. maxima* infection, alterations in the cecal microbiota persisted at 10 and 14 dpi (Fig. 5B; Fig. S3C). The top 15 bacterial genera accounted for 75.2%–87.4% of the total bacterial population in the cecum throughout infection, with *Faecalibacterium*, *Lactobacillus*, *Mediterraneibacter*, and *Ligilactobacillus* being the most prevalent (Fig. 5C; Table S2). Specifically, *Faecalibacterium*, represented mainly by two ASVs (F4 and F8), exhibited a progressive decline in response to *E. maxima* infection, while *Lactobacillus*, consisting primarily of group A *Lactobacillus* (F1), was enriched by *E. maxima* (Fig. 5D). *L. salivarius* (F2) also showed a significant increase in *E. maxima-*infected chickens at 5 and 7 dpi (Fig. 5D).

**Fig 6:**
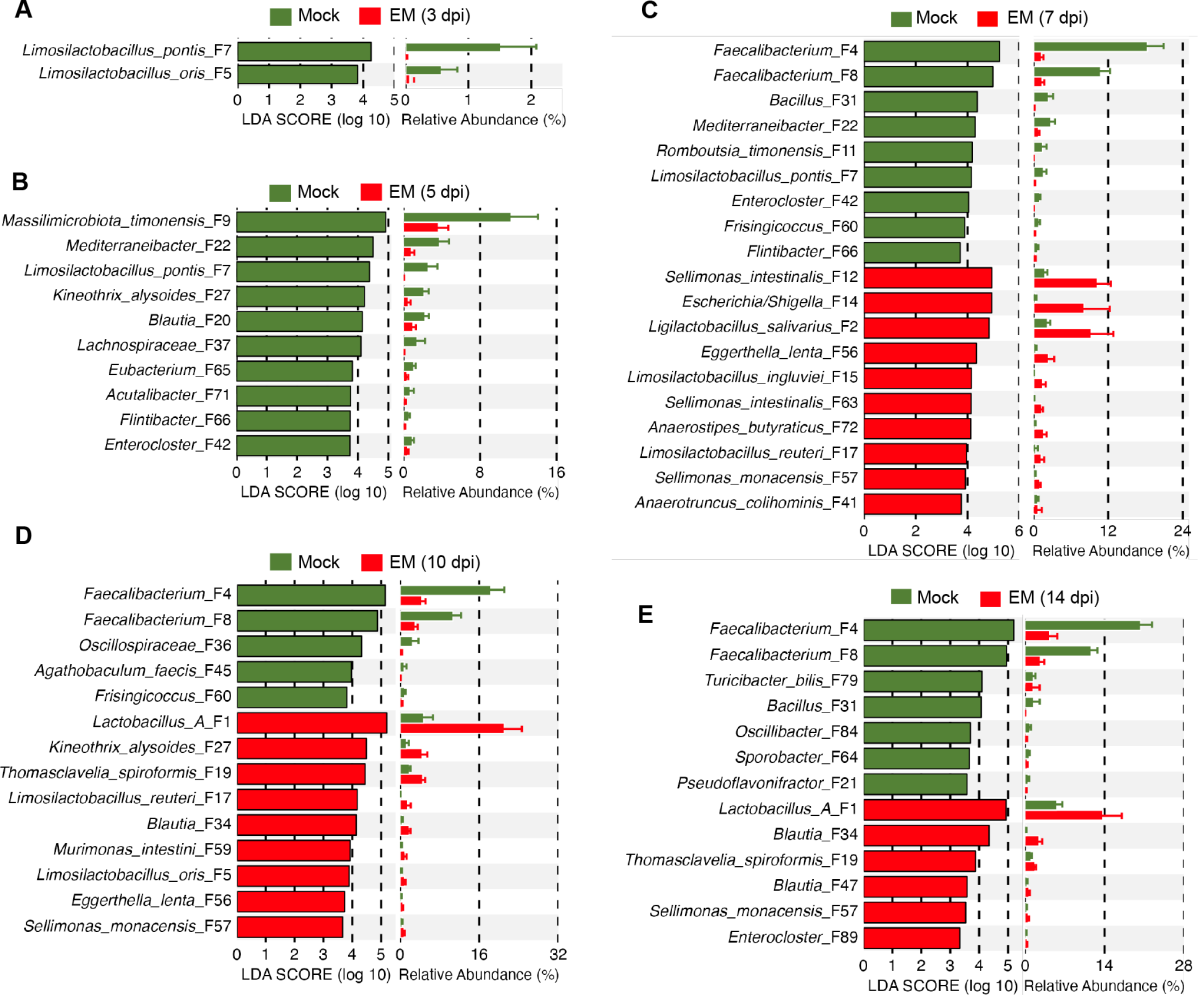
Differential enrichment of cecal bacteria in response to *E. maxima* (EM) infection. The cecal digesta samples were randomly collected from 10 chickens in either the mock or EM group at each of the five different days post-infection, followed by 16S rRNA gene sequencing and data analysis. LEfSe analysis was performed with the top 50 cecal bacterial ASVs between the mock and EM groups at 3 dpi (**A**), 5 dpi (**B**), 7 dpi (**C**), 10 dpi (**D**), and 14 dpi (**E**). The cut-off threshold was set at *P* < 0.05 and LDA score ≥ 3.0. Each panel shows the LDA score of differentially enriched ASVs on the left, while the relative abundances of these ASVs in the mock and EM groups are displayed on the right.

LEfSe analysis revealed significant alterations in the relative abundances of many cecal bacteria induced by *E. maxima* (Fig. S4; Fig. 6). It is particularly evident that *Faecalibacterium* was suppressed from 7 to 14 dpi, while *Ligilactobacillus* was enriched at 7 dpi, and *Lactobacillus* and *Limosilactobacillus* increased significantly at 10 dpi (Fig. S4). At the species level, *L. pontis* (F7) and *L. oris* (F5) were notably suppressed by *E. maxima* up to 7 dpi (Fig. 6A through C). The dominant *Faecalibacterium* (F4 and F8) was significantly diminished from 7 to 14 dpi in response to *E. maxima* infection, while *Escherichia* (F14), *L. salivarius* (F2), and *L. ingluviei* (F15) experienced transient blooms at 7 dpi (Fig. 6C through E). Several other lactic acid bacteria were also significantly enriched, albeit with different kinetics. For example, *L. reuteri* (F17) increased at 7 and 10 dpi, while group A *Lactobacillus* (F1) was enriched at 10 and 14 dpi. Several *Sellimonas* species (F12, F57, and F63) were also significantly enriched by *E. maxima* in the cecum at 7, 10, and/or 14 dpi (Fig. 6C through E).

**Fig 7:**
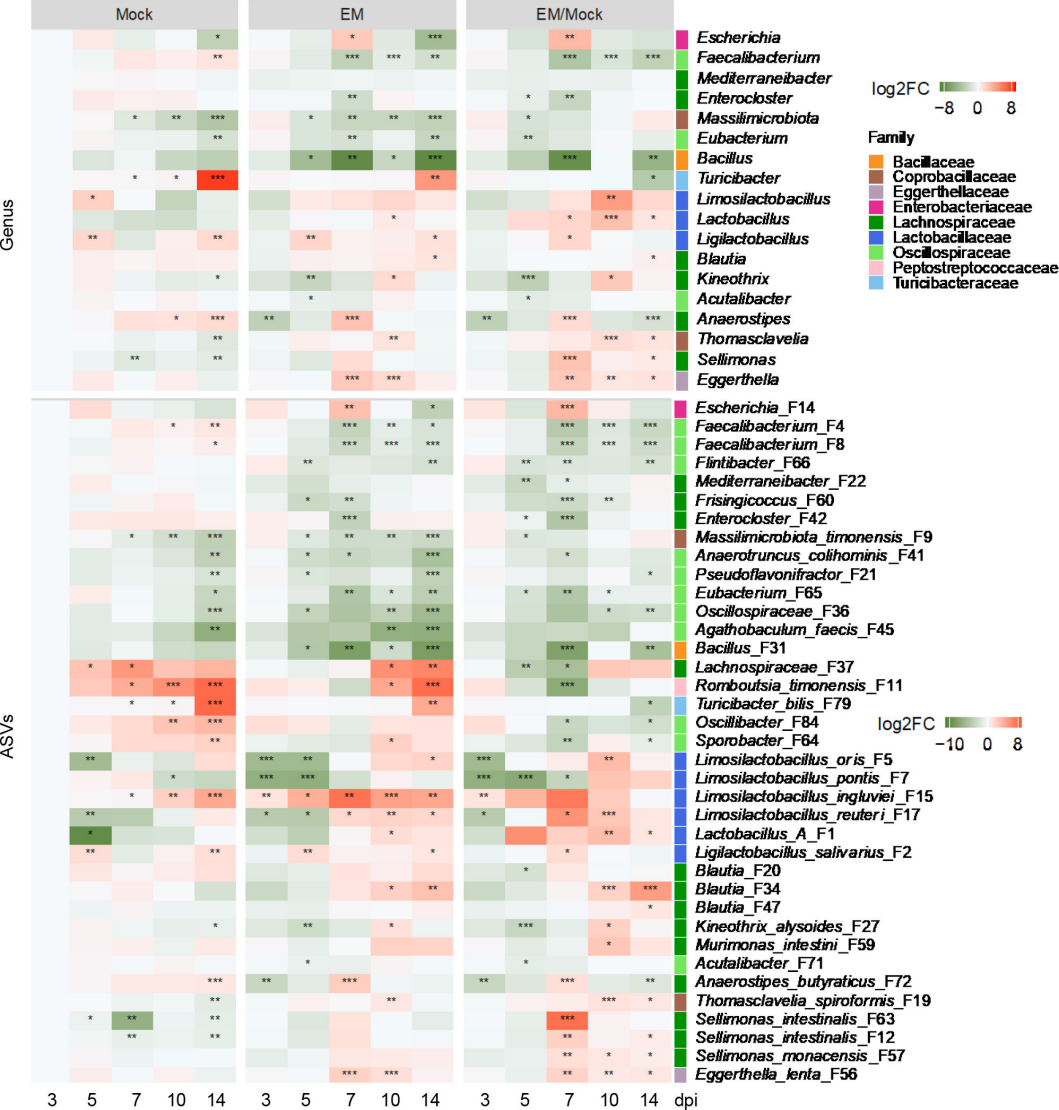
Dynamic patterns of differentially enriched cecal bacterial genera (top) and ASVs (bottom) in response to *E. maxima* (EM) infection. The left two panels of the heatmap depict log_2_ transformations of the fold changes of the differentially enriched bacteria in the ileum of mock and EM-infected chickens across five different days post-infection, relative to mock-infected animals at 3 dpi. *P < 0.05, **P < 0.01, and ***P < 0.001, relative to mock-infected animals at 3 dpi as determined by the Kruskal-Wallis test and *post hoc* Dunn’s test with Benjamini-Hochberg adjustment. The right panel illustrates log_2_ transformations of EM/mock fold differences of individual bacteria on each sampling day for the left two panels. *P < 0.05, **P < 0.01, and ***P < 0.001 between the mock and EM-infected animals on each sampling day as determined by Mann-Whitney *U* test with Benjamini-Hochberg adjustment.

Similar to the changes observed in ileal bacteria, differentially enriched cecal bacteria also displayed distinct temporal patterns in response to *E. maxima* infection (Fig. 7; Table S2). Along with a transient bloom of *Escherichia* at 7 dpi, *Lactobacillus, Limosilactobacillus*, and *Ligilactobacillus* also experienced enrichment after 5 dpi, while the most dominant *Faecalibacterium* was noticeably suppressed by *E. maxima* from 5 dpi and remained diminished until 14 dpi (Fig. 7). At the species level, both *Faecalibacterium* ASVs (F4 and F8) similarly declined between 7 and 14 dpi, along with multiple other SCFA producers, such as *Flintibacter* (F66), *Mediterraneibacter* (F22), *Frisingicoccus* (F60), *Enterocloster* (F42 and F89), and *Romboutsia timonensis* (F11).

Different species of *Lactobacillus, Limosilactobacillus*, and *Ligilactobacillus* were differentially regulated by *E. maxima* infection. Group A *Lactobacillus* (F1), *L. salivarius* (F2), and several *Limosilactobacillus* species (F5, F7, and F17) exhibited an initial decline at 3 dpi, followed by a quick recovery and enrichment between 5 and 10 dpi, before largely subsiding by 14 dpi. In contrast, *L. ingluviei* (F15) was consistently enriched by *E. maxima* during the initial 10 days of infection, peaking at 7 dpi. By 14 dpi, as the animals recovered from coccidiosis, *L. ingluviei* (F15) returned to normal healthy levels. Notably, a *Bacillus* species (F31) was particularly suppressed by *E. maxima* infection at 7 dpi, while several *Sellimonas* species (F12, F57, and F63) were enriched and peaked at 7 dpi (Fig. 7).

## DISCUSSION

*E. maxima* is not only a major cause of coccidiosis in chickens, but also a key predisposing factor for other diseases such as necrotic enteritis (2, 3, 13). This parasite preferentially targets the jejunal and ileal epithelia, causing damage to the intestinal lining, triggering an inflammatory response, and disrupting the intestinal microbiota (2, 3). These effects collectively lead to reduced growth performance, increased morbidity, and potential mortality. The pathogenicity of *E. maxima* is closely tied to its life cycle, which progresses through distinct phases. During the initial prepatent phase (0–3 dpi), after being ingested, sporulated oocysts release sporozoites that subsequently invade epithelial cells in the upper GIT, where they undergo three to four rounds of asexual reproduction to produce merozoites without causing significant damage to the host until the acute phase of infection (5–7 dpi), when the merozoites differentiate into male and female gametes, initiating sexual reproduction to form oocysts (14, 15). Shed in the feces, these oocysts sporulate under appropriate temperature and humidity, starting a new life cycle. Rapid replication of *E. maxima* within epithelial cells leads to rupture and extensive destruction of intestinal epithelial cells during 5–7 dpi, causing intestinal lesions and inflammation (14, 15). After 7 dpi, oocyst shedding in the feces rapidly declines, and the intestinal mucosa begins to repair and regenerate.

In this study, we examined microbiota alterations throughout the entire cycle of *Eimeria* infection, including the prepatent, acute, and recovery phases. We demonstrated a strong correlation between microbiota changes and the parasite’s life cycle. While no obvious changes in both the ileal and cecal microbiota were observed during the prepatent phase at 3 dpi, substantial alterations occurred during acute infection between 5 and 7 dpi, with the microbiota tending to normalize during the recovery phase beyond 7 dpi. This dysbiosis during acute infection likely stems from extensive intestinal epithelial cell destruction by *E. maxima* and subsequent inflammatory responses elicited. We also observed marked changes in the relative abundance of various intestinal bacteria in response to *E. maxima* infection. Most bacteria commonly residing in both the ileum and cecum exhibited similar patterns of differential enrichment in both GIT locations. Among the three dominant genera of lactic acid bacteria detected (*Lactobacillus, Ligilactobacillus*, and *Limosilactobacillus*), *Lactobacillus* was consistently enriched in both the ileum and cecum throughout the 14-day period following *E. maxima* infection. *Limosilactobacillus* showed an early decrease, followed by subsequent restoration and even enrichment in both locations during the recovery phase. However, *Ligilactobacillus* was largely suppressed in the ileum throughout the infection, but experienced enrichment at 7 dpi in the cecum.

Specific roles of different lactic acid bacteria in the GIT are largely unknown. An increase in *Lactobacillus* might act as a compensatory mechanism to repair epithelial damage, as *Lactobacillus* has been shown to strengthen the intestinal barrier (16). Similar enrichment of lactobacilli has been observed in patients with inflammatory bowel disease (17). In chickens co-infected with *E. maxima* and *Clostridium perfringens*, which induces necrotic enteritis, a similar increase in *Lactobacillus* has been documented (18). However, conflicting results have been reported in chickens infected with *E. tenella* (19, 20), possibly due to a lack of distinction among lactic acid bacteria, such as *Lactobacillus, Ligilactobacillus*, and *Limosilactobacillus,* in earlier microbiome analysis, the complex dynamics of different lactic acid bacteria in response to *E. maxima* infection, and variations in sampling time. Meta-analysis of these data may help clarify some of these discrepancies.

No studies have separated dominant lactic acid bacteria in the chicken GIT into different genera and species. Here, we resolved them to species levels in most cases. We were able to identify three *Lactobacillus* species (group A, *L. johnsonii*, and *L. kitasatonis*)*,* one *Ligilactobacillus* species (*L. salivarius*), and at least four species of *Limosilactobacillus* (*L. reuteri, L. ingluviei, L. oris*, and *L. pontis*). To our surprise, these dominant lactic acid bacterial species displayed distinct responses to *E. maxima* infection. Group A *Lactobacillus* and *L. johnsonii* were largely enriched in the ileum and cecum of infected chickens throughout the infection, while *L. kitasatonis* showed no obvious alterations in either the ileum or cecum during the 2-week infection period. *L. salivarius* experienced an obvious decline in the ileum at 10 dpi but a transient increase in the cecum at 7 dpi. Among the four *Limosilactobacillus* species, *L. oris*, *L. pontis,* and *L. reuteri* largely declined during acute infection, enriched at 10 dpi, and returned to normal during the recovery phase in both the ileum and the cecum; however, *L. ingluviei* showed different dynamics between the ileum and cecum. In the ileum, *L. ingluviei* markedly increased during early infection, before gradually declining by 10 dpi and returning to healthy levels by 14 dpi, while it progressively increased in the cecum during early infection, peaked at 7 dpi, and then gradually declined to normal levels at 14 dpi. Differential enrichment of these lactic acid bacteria may suggest their differential involvement in *E. maxima* infection. Given their known health-promoting properties (21), further investigation is warranted to determine the protective efficacy of individual lactic acid bacterial species against *Eimeria* infection.

*Faecalibacterium*, a predominant bacterium in the cecum, progressively declined during early infection, bottomed at 7 dpi, and remained diminished until 14 dpi. This decline in response to *Eimeria* infection has also been reported earlier (19, 22). Consistently, *Faecalibacterium* is a major colonic bacterium in humans and commonly reduced in inflammatory gastrointestinal disorders (23). As a SCFA-producing bacterium, *Faecalibacterium* possesses anti-inflammatory and barrier-protective properties (23), making it intriguing to study its role against *Eimeria* infection. Similarly, many other SCFA-producing bacteria, such as *Mediterraneibacter* and *Romboutsia timonensis*, also declined in response to *Eimeria* infection, suggesting the potential of supplementing SCFAs or SCFA-producing bacteria to alleviate coccidiosis.

Consistent with previous observations (9, 10), we noted a bloom of *Escherichia* in both the ileum and cecum at 7–10 dpi in response to *Eimeria* infection. In fact, many other intestinal inflammatory disorders are also associated with overgrowth of *E. coli* (24, 25). This transient increase is likely due to intestinal inflammation, which is associated with increased availability of reactive oxygen and nitrogen species that promote the growth of facultative and aerotolerant anaerobes like *E. coli* and *Staphylococcus*, as well as lactic acid bacteria such as *Lactobacillus*, *Ligilactobacillus*, *Limosilactobacillus*, and *Enterococcus*. Concurrently, obligate anaerobes such as *Faecalibacterium* and many other SCFA-producing bacteria are suppressed. Indeed, we observed transient blooms of individual species of *Lactobacillus*, *Ligilactobacillus*, and *Limosilactobacillus* in both the ileum and cecum, albeit with different kinetics. Additionally, *Enterococcus* and *Staphylococcus xylosus* increased in the ileum between 10 and 14 dpi of *E. maxima*. Increased prevalences of *E. coli*, *Enterococcus*, and *S. xylosus* are associated with intestinal inflammatory diseases in humans and animals (26, 27), consistent with the fact that *Eimeria* infection often leads to many secondary diseases.

However, the increased availability of reactive oxygen and nitrogen species does not fully account for the varied dynamic patterns observed among facultative and aerotolerant anaerobes during active inflammation. In this study, some bacteria peaked quickly during early infection, while others may peak during active infection or even during the resolution of infection. Additionally, while *S. xylosus* increased in response to *E. maxima* infection, another species of *Staphylococcus* (*S. gallinarum*) notably declined in the ileum at 7 dpi. Similarly, two other facultative members of the *Staphylococcaceae* family, *Mammaliicoccus lentus* and *Jeotgalicoccus aerolatus*, markedly declined in the ileum of *E. maxima*-infected chickens. The varied response of facultative and aerotolerant anaerobes to infection and inflammation is also reflected by their differing abilities to cope with nutrient changes, increased availability of mucin and mucin-derived sugars, and several other factors beyond their sensitivities to oxygen exposure in the GIT (24, 25).

Numerous studies have investigated the intestinal microbiota’s response to different *Eimeria* species during the acute phase of infection (5–7 dpi), with a majority concentrating on the impact of *E. tenella* or a mixture of multiple *Eimeria* species on the cecal microbiota (9, 10). However, none have examined microbiota alterations throughout the entire cycle of *Eimeria* infection, including the prepatent, acute, and recovery phases. Similar to our experimental design, two recent studies sampled the intestinal contents of chickens at multiple time points following *E. tenella* or *E. acervulina* infection and assessed the dynamic influence of either infection on alpha- and beta-diversities of intestinal microbiota (28, 29). However, these studies did not reveal a time-dependent response of the microbiota to infection. Specifically, they combined microbiome data across all time points and reported differentially enriched bacteria between control and infected chickens as a whole, rather than at each sampling point (28, 29).

We believe that understanding the dynamic changes in the intestinal microbiome throughout *Eimeria* infection is crucial for elucidating its pathogenesis and identifying potential disease biomarkers. Major differentially enriched bacteria identified in this study, such as *Faecalibacterium* and various species of lactic acid bacteria (*Lactobacillus*, *Ligilactobacillus*, and *Limosilactobacillus*), could be investigated for their roles in the progression of coccidiosis. Additionally, these bacteria could be targeted to develop effective microbiome-based strategies to mitigate coccidiosis and potentially other diseases. Furthermore, bacteria that are altered early during infection may be explored for the early diagnosis of coccidiosis.

It is noteworthy that the growth performance of *E. maxima*-infected chickens did not fully recover by 14 dpi due to a lag in compensatory growth. Continuing the analysis of intestinal microbiota until infected chickens reach the body weight of healthy ones would provide valuable insights. Understanding whether the intestinal microbiota, particularly the cecal microbiota, fully returns to normal or experiences sustained alterations in response to *Eimeria* infection is crucial. However, the failure of animal growth performance to fully recover by 14 dpi may also be attributed to the reinfection of chickens by *E. maxima* through the recycling of oocysts. A portion of oocysts shed onto wood shavings between 5 and 7 dpi in our experimental setting could sporulate within 2–3 days, potentially leading to reinfection of chickens. Consequently, *E. maxima*-infected chickens might have experienced a second round of mild infection by 14 dpi, which could explain why the intestinal microbiota, particularly the cecal microbiota, was not fully restored to healthy levels. Indeed, a few cecal bacteria, such as *Faecalibacterium*, appeared to experience a biphasic decline between 7 and 14 dpi in our trial. To minimize oocyst recycling, it is advisable to house animals in battery cages or replace old bedding material with fresh material after 7 dpi for this type of animal experiment in the future.

### Conclusions

In summary, *E. maxima* infection triggers intestinal dysbiosis, coinciding with the life cycle of *Eimeria*. Minimum changes in the intestinal microbiota occur during early infection, while drastic alterations take place during acute infection. Upon recovery from the infection, the intestinal microbiota tends to return to a healthy state. Along with the initiation of intestinal inflammation, facultative and aerotolerant bacteria such as *Escherichia*, *Enterococcus*, *Lactobacillus*, *Ligilactobacillus*, and *Limosilactobacillus* largely experience transient blooms, although with varying kinetics. Additionally, different species of *Lactobacillus*, *Ligilactobacillus*, and *Limosilactobacillus* exhibit distinct dynamic patterns in response to *E. maxima* infection. Concurrently, a variety of SCFA-producing bacteria such as *Faecalibacterium* are reduced by *E. maxima*. These findings shed light on the influence of *Eimeria* infection on the intestinal microbiota and suggest the potential for microbiome-based strategies in the control and prevention of coccidiosis.

## MATERIALS AND METHODS

### Chicken model of coccidiosis

A total of 120 day-of-hatch unvaccinated male Cobb broiler chicks were obtained from Cobb-Vantress Hatchery (Siloam Springs, AR, USA) and housed in floor pens covered with fresh pine wood shavings in an environmentally controlled room under standard management as recommended by Cobb-Vantress. Chickens were maintained in 10 pens with 12 chickens/pen, with free access to tap water and mash corn-soybean meal standard starter diet (21% crude protein) throughout the trial. After overnight fasting, chickens in five pens were orally inoculated on day 10 with 2 × 10^4^ sporulated oocysts of *E. maxima* strain M6 (kindly provided by John R. Barta, University of Guelph, Canada) in 1 mL saline, while the animals in the remaining five pens were inoculated with 1 mL saline alone as the mock-infected group. Animals were observed daily for clinical signs of coccidiosis, such as lethargy, bloody diarrhea, and ruffled feathers. At 3, 5, 7, 10, and 14 dpi, 10 birds from each group were randomly selected with two birds/pen, weighed, and euthanized by CO_2_ asphyxiation. Approximately 0.2 and 0.5 g of the digesta were collected aseptically from the cecum and proximal ileum, respectively, snap-frozen in liquid nitrogen, and stored at −80°C until use. The pens for two groups of chickens were separated with plastic sheets, and extreme cautions were exercised to minimize cross-contamination during animal care and sampling. Mock-infected animals were always handled first, and thorough sanitization procedures were applied when handling animals between treatments.

### Bacterial DNA isolation and 16S rRNA gene sequencing

Fecal DNA MicroPrep and MiniPrep Kits (Zymo Research Irvine, CA, USA) were used for the isolation of DNA from the ileal and cecal digesta, respectively. The concentration and quality of DNA were measured by Nanodrop One Spectrophotometer (Thermo Fisher Scientific). High-quality DNA samples were shipped on dry ice to Novogene (Beijing, China) for PE250 deep sequencing of the V3–V4 region of bacterial 16S rRNA gene using primers (341F: CCTAYGGGRBGCASCAG and 806R: GGACTACNNGGGTATCTAAT) on an Illumina platform. PCR amplification and library preparation were performed by Novogene (Beijing, China) using NEBNext Ultra Library Prep Kit (New England Biolabs, Ipswich, MA, USA).

### Bioinformatics and statistical analysis

Downstream bioinformatic analysis was conducted as we previously described (30–32). Briefly, raw sequencing reads were analyzed in QIIME 2 v2023.7 (33). Adapter, barcode, and primer sequences were removed before downstream analysis using the “cutadapt.” Paired-end reads were then merged using “vsearch join-pairs,” and low-quality reads were filtered out using “quality-filter q-score.” Sequences were trimmed to 402 nucleotides and denoised using Deblur (34). The resulting sequences were then classified into bacterial ASVs using the RDP 16S rRNA training set (version 18) and Bayesian classifier. A bootstrap confidence of 80% was used for taxonomic classification. ASVs with a classification confidence of <80% were assigned the name of the last confidently assigned level followed by “_unclassified.” ASVs appearing in <5% of samples were removed from analysis. Top 100 ASVs and all differentially enriched bacteria were further confirmed and reclassified, if necessary, based on a more recent EzBioCloud 16S database (v2023.08.23) (35). The read counts table was normalized using cumulative sum scaling in the metagenomeSeq package of R version 1.43.0 (36).

Analysis and visualization of α- and β-diversities of the microbiota composition were conducted in R v4.3.2 (37), utilizing the “phyloseq” package v1.46.0 (38). To visualize the overall biodiversity and complexity within samples, the number of ASVs, Pielou’s evenness index, and Shannon index were used to calculate and display the richness, evenness, and overall diversity. The β-diversity was determined using weighted and unweighted UniFrac distances. Statistical significance in α-diversity and relative abundance for each sampling day was determined using the non-parametric Mann-Whitney *U* test. Significance in β-diversity was determined using non-parametric permutational multivariate analysis of variance with 999 permutations using the vegan package v. 2.6.4. *P* < 0.05 was considered statistically significant. Differential enrichment of bacterial ASVs between groups was determined using LEfSe (12), with the all-against-all multiclass analysis using *P* < 0.05 and a logarithmic LDA score of ≥3.0 as the threshold. Fold changes in relative abundances of identified differentially enriched bacterial ASVs were further calculated relative to mock-infected animals at 3 dpi, followed by log2 transformation and visualization in a heatmap.

## Data Availability

The raw sequencing reads of this study have been deposited in the NCBI Sequence Read Archive (SRA) database under BioProject PRJNA1076950.

## SUPPLEMENTAL MATERIAL

Supplemental tables and figures spectrum.00823-24-s0001.pdf Tables S1 and S2; Fig. S1 to S4.

ASM does not own the copyrights to Supplemental Material that may be linked to, or accessed through, an article. The authors have granted ASM a non-exclusive, world-wide license to publish the Supplemental Material files. Please contact the corresponding author directly for reuse.
