# Supplementary material for: Dynamic response of the intestinal microbiome to Eimeria maxima-induced coccidiosis in chickens
Source: Microbiol Spectr. 2024 Sep 9;12(10):e00823-24. doi: 10.1128/spectrum.00823-24 (PMC11448223; doi:10.1128/spectrum.00823-24)
Supplement: Supplemental tables and figures — Tables S1 and S2; Fig. S1 to S4. [file spectrum.00823-24-s0001.pdf]

Supplemental Information

Table S1. Relative abundances and fold changes of the ileal bacteria in response to *E. maxima* infection[a]

Taxon	3 dpi			5 dpi			7 dpi			10 dpi			14 dpi		
	Mock (%)	EM (%)	FC[b]	Mock (%)	EM (%)	FC	Mock (%)	EM (%)	FC	Mock (%)	EM (%)	FC	Mock (%)	EM (%)	FC
**Genera**															
Lactobacillus	41.13	70.37	1.71	38.37	83.9**	2.19	23.51	71.3***	3.03	12.82	25.96**	2.03	18.29	26.28	1.44
Limosilactobacillus	35.4	9.82***	0.28	33.99	7.52**	0.22	14.15	10.49	0.74	24.68	20.05	0.81	18.30	17.22	0.94
Ligilactobacillus	11.17	5.74	0.51	2.64	2.24	0.85	23.18	7.04	0.30	45.66	8.08***	0.18	37.67	23.89	0.63
Corynebacterium	0.89	2.97	3.33	5.01	1.11**	0.22	7.54	1.79	0.24	3.69	2.06	0.56	4.58	5.32	1.16
Enterococcus	0.93	0.75	0.81	0.18	0.28	1.53	0.74	0.13**	0.18	2.04	7.75**	3.80	0.40	0.62	1.54
Staphylococcus	0.04	0.03	0.87	0.15	0.02	0.13	0.55	0.06**	0.12	0.22	0.73	3.28	0.18	1.95	10.91
Escherichia	0.05	0.05	1.11	0.02	0.03	1.12	0.02	0.09**	4.12	0.03	4.77**	145.3	0.02	0.02	1.00
Brachybacterium	0.01	0.02	1.74	0.09	0.008	0.09	0.49	0.008**	0.02	0.16	0.09	0.56	1.04	0.10	0.10
Aerococcus	0.03	0.18	6.79	0.04	0.01	0.37	0.11	0.01*	0.11	0.07	0.05	0.69	0.08	0.04	0.51
Jeotgalicoccus	0.01	0.01	0.96	0.06	0.004	0.06	0.12	0.008*	0.07	0.08	0.02	0.23	0.17	0.04	0.25
Rothia	0.004	0.05**	11.88	0.005	0.007	1.50	0.004	0.004	0.99	0.009	0.005	0.54	0.006	0.005	0.84
Mammaliicoccus	0.008	0.007	0.92	0.009	0.007	0.84	0.07	0.009**	0.12	0.04	0.03	0.44	0.10	0.03	0.34
Paracoccus	0.01	0.04	3.60	0.02	0.01	0.60	0.01	0.02	1.12	0.02	0.01	0.88	0.04	0.03	0.69
Kocuria	0.004	0.005*	1.30	0.004	0.004	0.81	0.004	0.004	0.99	0.005	0.004	0.87	0.004	0.004	0.97
**ASVs**															
Lactobacillus_A_F1	30.57	29.24	0.96	15.53	21.30	1.37	12.66	46.74	3.69	1..04	30.81	2.79	22.38	30.81	1.38
Ligilactobacillus_salivarius_F2	10.40	12.87	1.23	3.55	3.19	0.90	22.39	13.87	0.62	46.27	12.60***	0.27	38.86	26.52	0.68
Lactobacillus_kitasatonis_F3	17.74	31.25	1.76	22.04	53.18	2.41	9.23	16.83	1.83	0.73	1.03	1.41	0.92	0.23	0.25
Faecalibacterium_F4	0.008	0.007	0.90	0.010	0.114	10.94	0.01	0.03	1.93	0.01	0.01	1.00	0.01	0.005	0.52
Limosilactobacillus_oris_F5	12.35	2.44**	0.19	3.47	0.44**	0.13	10.59	2.98	0.28	4.86	13.91*	2.86	8.69	8.94	1.03
Cuneatibacter_F6	0.03	0.08	2.59	0.04	0.04	1.00	0.14	0.07	0.53	0.15	0.13	0.91	0.05	0.06	1.20
Limosilactobacillus_pontis_F7	13.21	0.31**	0.02	13.53	0.32**	0.02	8.46	1.61	0.19	3.30	2.71	0.82	4.82	3.85	0.80
Faecalibacterium_F8	0.01	0.008	0.72	0.01	0.08	6.05	0.02	0.04	1.79	0.01	0.009	0.91	0.01	0.004	0.39
Massilimicrobiota_timonensis_F9	0.017	0.015	0.85	0.03	0.02	0.59	0.009	0.007	0.75	0.03	0.01	0.49	0.007	0.007	0.91
Limosilactobacillus_reuteri_F10	0.46*	3.88	8.40	19.16	5.33	0.28	0.55	0.79	1.42	0.01	0.25	19.41	0.004	0.004	0.92
Romboutsia_timonensis_F11	0.19	0.04	0.22	0.02	0.08	4.28	4.65	0.02	0.005	0.04	0.03	0.76	0.84	0.08	0.10
Sellimonas_intestinalis_F12	0.02	0.01	0.50	0.02	0.12	6.57	0.01	0.07	5.49	0.03	0.09*	3.01	0.03	0.04	1.46
Corynebacterium_stationis_F13	0.63	1.65	2.61	4.23	0.86	0.20	5.30	2.13	0.40	2.63	2.46	0.93	2.94	4.13	1.40
Escherichia_F14	0.31	0.28	0.89	0.04	0.04	0.96	0.04	0.43**	12.07	0.05	5.71**	116.5	0.02	0.03	1.31
Limosilactobacillus_ingluviei_F15	0.02	2.93	114.2	0.20	0.39	1.97	0.80	1.55	1.95	10.67	2.49*	0.23	4.04	2.58	0.64
Lactobacillus_johnsonii_F16	2.42	1.57	0.64	1.41	8.26	5.86	1.42	3.13	2.21	0.31	2.65**	8.42	1.11	1.44	1.30
Limosilactobacillus_reuteri_F17	2.03	1.40	0.69	0.048	0.003	0.078	1.49	2.45	1.50	1.29	3.85	2.99	1.82	3.88	2.14
Mediterraneibacter_F18	0.018	0.022	1.22	0.11	0.01	0.13	0.04	0.27	7.09	0.03	0.008	0.24	0.02	0.009	0.48
Thomasclavelia_spiroformis_F19	0.007	0.006	0.77	0.03	0.11	3.74	0.008	0.02	2.25	0.02	0.03	1.18	0.01	0.02	1.42
Blautia_F20	0.027	0.016	0.57	0.13	0.13	0.97	0.05	0.16	3.01	0.06	0.18	3.09	0.03	0.06	1.80

Enterococcus_durans/hirae_F23	3.69	1.30	0.35	1.58	0.30	0.19	1.37	0.23*	0.17	0.98	3.73	3.80	0.33	1.89	5.73
Staphylococcus_gallinarum_F25	0.25	0.09	0.35	2.04	0.08	0.04	6.32	0.06*	0.01	3.28	0.75	0.23	0.92	0.89	0.97
Corynebacterium_stationis_F28	0.28	1.07*	3.70	1.87	0.44	0.24	2.45	1.05	0.43	1.50	1.21	0.81	1.30	1.83	1.42
Enterococcus_cecorum_F30	0.06	0.17	2.88	0.06	0.11	1.83	0.40	0.03	0.07	2.53	8.00	3.15	0.50	0.29	0.58
Corynebacterium_casei_F32	0.22	0.55	2.51	1.51	0.33*	0.22	2.09	0.76	0.36	1.23	0.87	0.70	1.25	1.53	1.23
Staphylococcus_xylosus_F80	0.005	0.012	2.26	0.005	0.04	6.45	0.006	0.04	6.77	0.008	0.41*	52.0	0.004	2.64*	591.9

[a] Day-10 male broiler chickens were either challenged with 2×10^4 sporulated oocysts of *Eimeria maxima* (EM) or mock-infected with saline. At 3, 5, 7, 10, and 14 days post-infection (dpi), 10 chickens from each group were randomly euthanized to collect the ileal digesta samples for 16S rRNA gene sequencing. Average relative abundances (%) of the top 15 bacterial genera and the top 20 amplicon sequence variants (ASVs), as well as a few selected ASVs, in the ileum across five different dpi are shown. Pairwise Mann-Whitney U test was performed for each genus and ASV, and statistical significance was further adjusted with false discovery rate (FDR). *FDR < 0.05, **FDR < 0.01, and **FDR < 0.001.

[b] Fold change (FC) was calculated by comparing the relative abundance of each genus or ASV in the EM group to that in the mock group.

Table S2. Relative abundances and fold changes of the cecal bacteria in response to *E. maxima* infection[a]

Taxon	3 dpi			5 dpi			7 dpi			10 dpi			14 dpi		
	Mock (%)	EM (%)	FC[b]	Mock (%)	EM (%)	FC	Mock (%)	EM (%)	FC	Mock (%)	EM (%)	FC	Mock (%)	EM (%)	FC
**Genera**															
Lactobacillus	10.58	5.43	0.51	4.38	12.2	2.74	2.91	9.49	3.26	2.52	17.4***	6.92	5.99	11.80	1.97
Mediterraneibacter	7.69	4.95	0.64	8.93	5.31	0.60	8.97*	6.10	0.68	8.52	5.49	0.64	6.07	6.23	1.03
Ligilactobacillus	3.16	2.23	0.71	9.54	10.48	1.10	1.39	4.36*	3.14	4.58	4.79	1.04	9.17	7.05	0.77
Faecalibacterium	13.51	16.1	1.19	11.17	6.48	0.58	24.96	1.83***	0.07	19.88	6.74***	0.34	27.59	3.54***	0.13
Blautia	4.18	2.86	0.68	5.82	5.91	1.02	5.23	5.29	1.01	6.23	6.76	1.08	6.11	8.59*	1.41
Massilimicrobiota	6.94	10.49	1.51	8.03	2.49**	0.31	3.04	1.15	0.38	1.38	1.20	0.87	0.57	0.91	1.66
Sellimonas	4.31	3.40	0.79	3.32	1.80	0.54	1.78	11.2**	6.27	2.80	3.89	1.39	2.09	3.61*	1.72
Limosilactobacillus	1.92	0.51	0.26	7.60	3.15	0.41	1.89	4.15	2.19	0.28	5.99***	21.24	1.17	3.51	3.00
Thomasclavelia	1.95	1.70	0.87	2.05	2.80	1.37	1.33	2.23	1.69	1.61	4.14**	2.58	0.81	1.67*	2.07
Escherichia	0.41	0.44	1.07	0.69	0.23	0.35	0.24	2.01**	8.21	0.40	0.19	0.46	0.05	0.02	0.33
Kineothrix	1.10	0.39	0.35	1.54	0.18	0.12	0.84	0.42	0.51	0.58	2.97***	5.13	0.63	0.79	1.26
Bacillus	1.47	0.74	0.50	0.54	0.06	0.12	1.15	0.005**	0.00	0.26	0.25	0.94	0.17	0.005**	0.03
Eisenbergiella	0.90	0.60	0.67	0.92	0.33	0.36	0.72	0.57	0.81	0.74	0.33*	0.44	0.61	0.30	0.49
Enterocloster	1.22	1.13	0.92	2.01	1.12*	0.56	1.68*	0.29	0.17	1.73	1.52	0.88	1.19	1.18	0.99
Eubacterium	0.94	1.10	1.17	1.18	0.34*	0.29	0.67	0.28	0.42	0.62	0.66	1.06	0.28	0.16	0.58
Anaerostipes	0.16	0.02	0.12	0.18	0.08	0.43	0.37	1.01*	2.72	0.36	0.15	0.40	0.45	0.13	0.30
Turicibacter	0.004	0.005	1.17	0.005	0.004	0.90	0.004	0.004	0.99	0.005	0.005	0.93	1.12*	0.10	0.09
Eggerthella	0.19	0.21	1.11	0.32	0.19	0.58	0.20	0.96*	4.88	0.28	0.54	1.91	0.13	0.27	2.12
Acutalibacter	0.26	0.23	0.88	0.32	0.15*	0.48	0.27	0.14	0.54	0.35	0.35	1.00	0.23	0.24	1.04
**ASVs**															
Lactobacillus_A_F1	5.12	1.00	0.20	0.005	0.34	71.0	1.16	6.38	5.51	1.41	16.59**	11.75	5.23	11.64*	2.22
Ligilactobacillus_salivarius_F2	3.13	2.20	0.70	9.50	10.43	1.10	1.36	4.33**	3.19	4.55	4.75	1.04	0.13	7.01	0.77
Lactobacillus_kitasatonis_F3	0.98	1.02	1.04	3.28	6.40	1.95	0.97	0.49	0.50	0	0	0	0	0	0
Faecalibacterium_F4	8.47	9.78	1.15	7.40	4.09	0.55	15.88	0.84**	0.05	12.17	3.91***	0.32	17.69	2.22***	0.13
Limosilactobacillus_oris_F5	0.24	0.005**	0.20	0.005	0.005	0.94	0.14	0.26	1.80	0.07	0.90**	12.74	0.77	1.13	1.48
Cuneatibacter_F6	4.18	2.06	0.49	5.13	5.02	0.98	7.54	2.80	0.37	7.80	1.44	0.18	6.07	12.16	2.00
Limosilactobacillus_pontis_F7	0.47	0**	0	0.67	0.004***	0.006	0.23	0.66*	2.93	0.08	0.67	8.65	0.05	0.27	5.11
Faecalibacterium_F8	6.04	6.11	1.01	4.31	2.38	0.55	8.80	0.84**	0.10	7.21	2.54***	0.35	9.89	1.66***	0.17
Massilimicrobiota_timonensis_F9	7.09	10.75	1.52	8.03	2.49**	0.31	3.04	1.15	0.38	1.38	1.20	0.87	0.55	0.91	1.66
Limosilactobacillus_reuteri_F10	0.05	0.17	3.45	3.24	2.27	0.70	0.09	0.15	1.69	0	0	0	0	0	0
Romboutsia_timonensis_F11	0.03	0.07	2.72	0.21	0.05	0.24	0.67	0.004**	0.007	2.07	0.78	0.38	7.83	7.45	0.95
Sellimonas_intestinalis_F12	3.20	2.84	0.87	2.51	1.21	0.48	1.43	8.58*	5.97	2.07	2.91	1.40	1.57	2.85	1.81
Escherichia_F14	0.19	0.44	2.30	0.63	0.18	0.30	0.14	2.00**	14.44	0.11	0.18	1.65	0.05	0.01	0.30
Limosilactobacillus_ingluviei_F15	0.004	0.009	2.41	0.005	0.08	17.2	0.005	0.79*	157.4	0.02	0.14	6.46	0.10	0.11	1.07
Lactobacillus_johnsonii_F16	0.01	0.008	0.87	0.06	0.16	2.44	0.07	0.18	2.57	0.01	0.02	1.79	0.01	0.02	1.23
Limosilactobacillus_reuteri_F17	0.08	0.005	0.06	0.005	0.004	0.90	0.005	0.31**	61.0	0.05	0.49***	7.88	0.15	0.34	2.23
Mediterraneibacter_F18	0.15	0	0	0.76	0.11	0.15	0	0	0	0.88	0.07***	0.08	1.17	0.05	0.04

Thomasclavelia_spiroformis_F19	1.94	1.70	0.87	2.05	2.80	1.37	1.31	2.22	1.69	1.60	4.14***	2.59	0.80	1.66**	2.08
Blautia_F20	0.92	0.32	0.35	2.04*	0.46	0.23	1.22	2.28	2.04	1.56	1.46	0.94	2.56	2.95	1.15
Enterococcus_durans/hirae_F23	0.05	0.05	1.04	0.10	0.05	0.47	0.02	0.03	1.61	0.02	0.01	0.97	0.01	0.03	3.67
Bacillus_F31	1.49	0.75	0.50	0.54	0.06	0.24	1.15**	0.005	0.004	0.26	0.25	0.94	0.17	0.005	0.03

[a] Day-10 male broiler chickens were either challenged with 2×10^4 sporulated oocysts of *Eimeria maxima* (EM) or mock-infected with saline. At 3, 5, 7, 10, and 14 days post-infection (dpi), 10 chickens from each group were randomly euthanized to collect the cecal digesta samples for 16S rRNA gene sequencing. Average relative abundances (%) of the top 20 bacterial genera and the top 20 amplicon sequence variants (ASVs), as well as two selected ASVs, in the cecum across five different dpi are shown. Pairwise Mann-Whitney U test was performed for each genus and ASV, and statistical significance was further adjusted with false discovery rate (FDR). *FDR < 0.05, **FDR < 0.01, and **FDR < 0.001.

[b] Fold change (FC) was calculated by comparing the relative abundance of each genus or ASV in the EM group to that in the mock group.

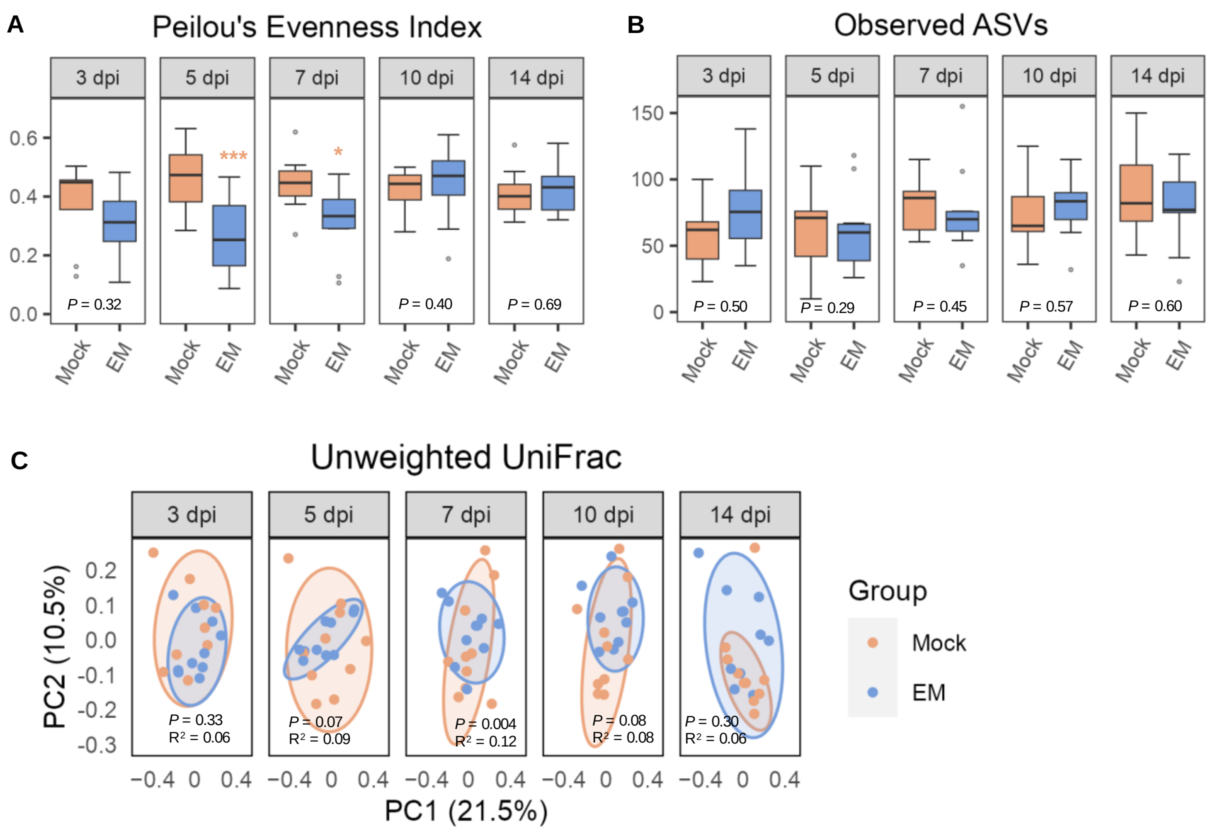

Fig. S1. **Diversity of the ileal microbiota in response to *E. maxima* (EM) infection.** Ten ileal digesta samples were randomly collected from either mock or EM-infected chickens at each of the five different days post-infection (dpi), followed by 16S rRNA gene sequencing and data analysis. (**A**) Pielou's evenness index and (**B**) Observed ASVs shown in box and whisker plots. Each box indicates the median, 25th and 75th percentiles, while whiskers extend to 1.5 interquartile range. $*P < 0.05$, $***P < 0.001$ as determined by Mann-Whitney U test. (**C**) Principal coordinates analysis (PCoA) plots displaying unweighted UniFrac distances, with each dot representing an individual ileal digesta sample. Statistical significance was determined using PERMANOVA with 999 permutations.

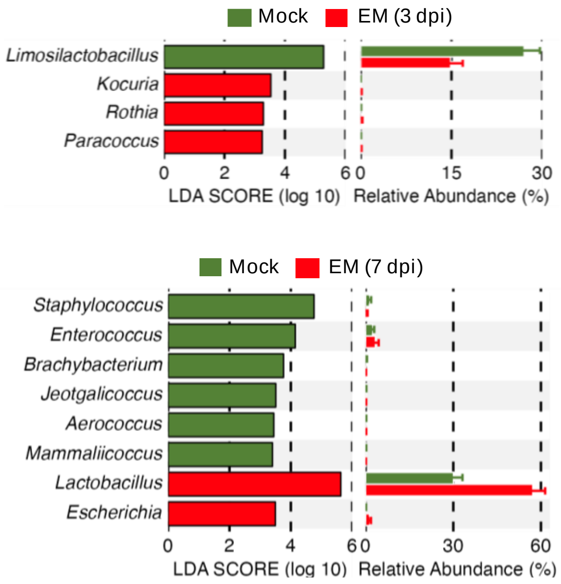

Fig. S2. **Differential enrichment of ileal bacteria in response to *E. maxima* (EM) infection.** Ten ileal digesta samples were randomly collected from either mock or EM-infected chickens at each of the five different days post-infection (dpi), followed by 16S rRNA gene sequencing and data analysis. LEfSe analysis was performed with the top 15 ileal bacterial genera between the mock and EM groups at 3, 5, 7, 10, and 14 dpi. The cut-off threshold was set at $P < 0.05$ and LDA score ≥ 3.0. Each panel shows the LDA score of differentially enriched genera on the left, while the relative abundances of these genera in the mock and EM groups are displayed on the right. Note that no differentially enriched bacteria were identified at 14 dpi.

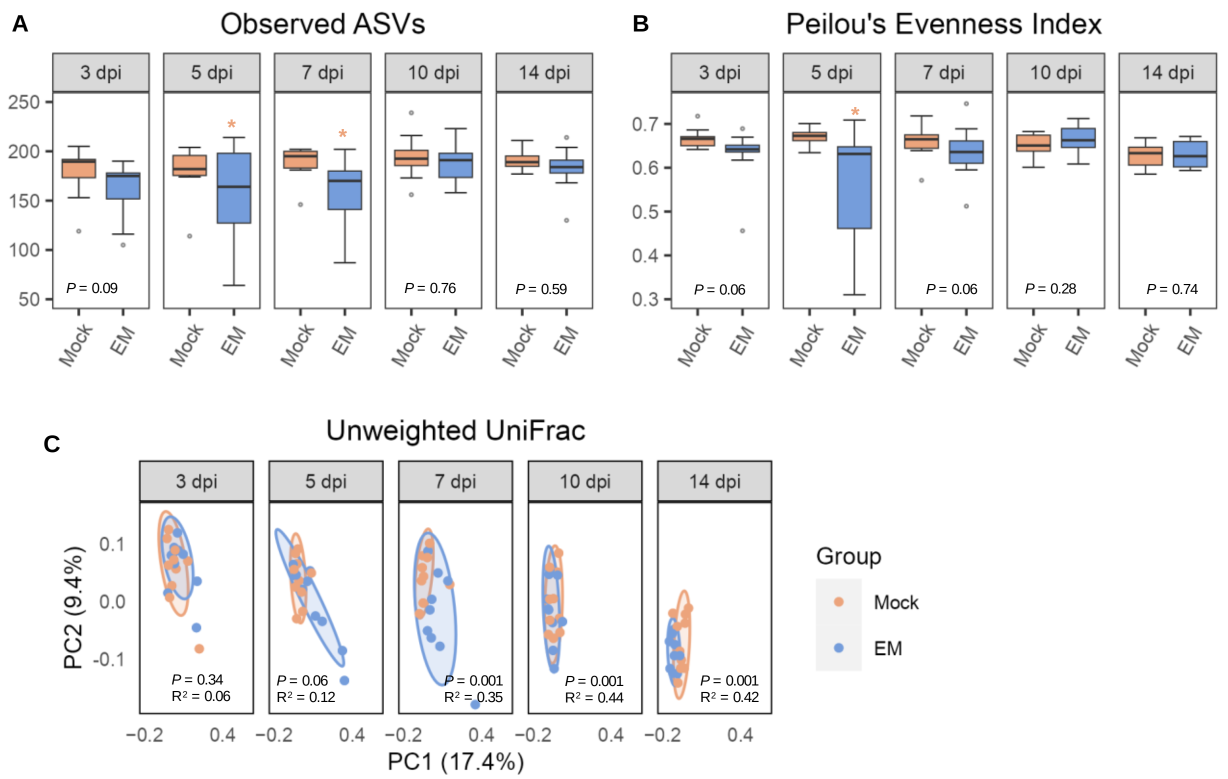

Fig. S3. **Diversity of the cecal microbiota in response to *E. maxima* (EM) infection.** Ten cecal digesta samples were randomly collected from either mock or EM-infected chickens at each of the five different days post-infection (dpi), followed by 16S rRNA gene sequencing and data analysis. (**A**) Observed ASVs and (**B**) Pielou's evenness index shown in box and whisker plots. Each box indicates the median, 25th and 75th percentiles, while whiskers extend to 1.5 interquartile range. *P < 0.05 as determined by Mann-Whitney U test. (**C**) Principal coordinates analysis (PCoA) plots displaying unweighted UniFrac distances, with each dot representing an individual cecal digesta sample. Statistical significance was determined using PERMANOVA with 999 permutations.

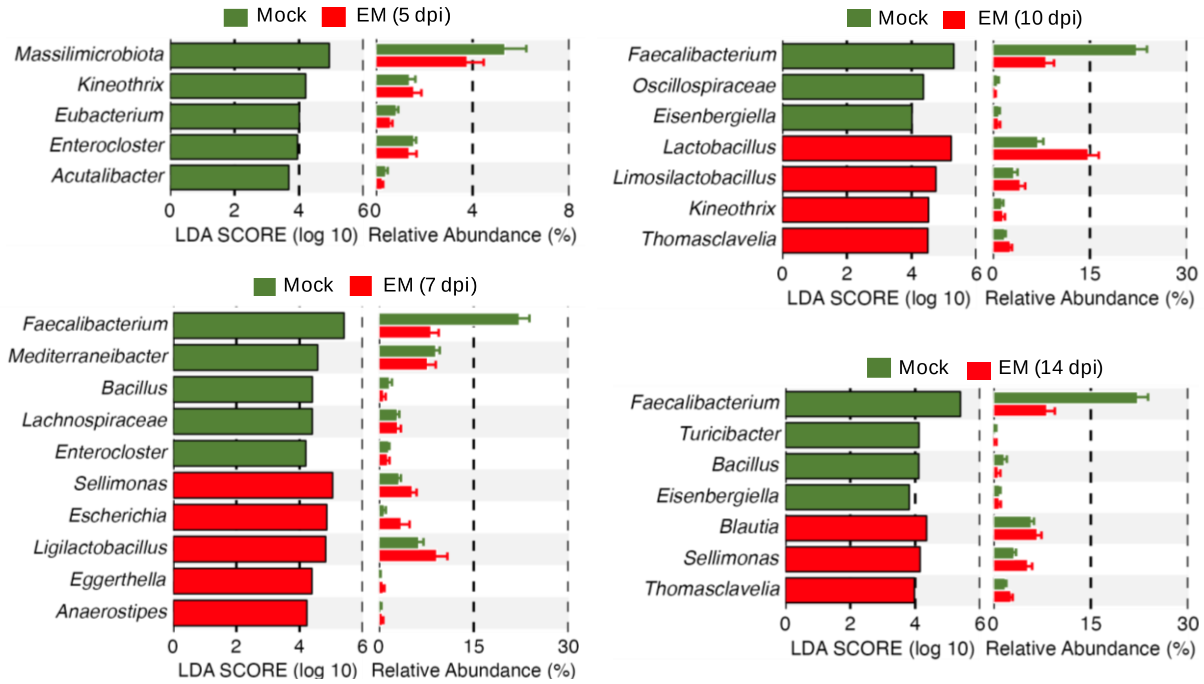

Fig. S4. **Differential enrichment of cecal bacteria in response to *E. maxima* (EM) infection.** Ten cecal digesta samples were randomly collected from either mock or EM-infected chickens at each of the five different days post-infection (dpi), followed by 16S rRNA gene sequencing and data analysis. LEfSe analysis was performed with the top 20 cecal bacterial genera between the mock and EM groups at 3, 5, 7, 10, and 14 dpi. The cut-off threshold was set at $P < 0.05$ and LDA score ≥ 3.0. Each panel shows the LDA score of differentially enriched genera on the left, while the relative abundances of these genera in the mock and EM groups are displayed on the right. Note that no differentially enriched bacteria were identified at 3 dpi.
